# Langzeitverläufe bipolarer Störungen

**DOI:** 10.1007/s00115-024-01791-6

**Published:** 2024-12-21

**Authors:** Tabea Czempiel, Pavol Mikolas, Michael Bauer, Sabrina Vogel, Philipp Ritter

**Affiliations:** Klinik und Poliklinik für Psychiatrie und Psychotherapie, Universitätsklinikum Carl Gustav Carus, Technischen Universität Dresden, Fetscherstraße 74, 01307 Dresden, Deutschland

**Keywords:** Subtyp, Psychosoziales Funktionsniveau, Strukturelle Hirnbildgebung, Biomarker, Langzeitprognose, Subtype, Psychosocial functional level, Structural brain imaging, Biomarkers, Long-term prognosis

## Abstract

**Hintergrund:**

Die bipolare Störung (englisch: „bipolar disorder“, kurz: BD) ist eine schwerwiegende Erkrankung mit sehr heterogenen Verlaufsformen. Während ein Teil der Patienten keine oder kaum langfristige Beeinträchtigungen aufweist, zeigt ein anderer Teil der Betroffenen erhebliche neurokognitive Einschränkungen mit deutlichem psychosozialen Funktionsabbau. Welche Faktoren den Krankheitsverlauf beeinflussen, ist Gegenstand aktueller Forschungsbestrebungen.

**Ziel der Arbeit:**

In dieser Übersichtsarbeit werden der Langzeitverlauf der bipolaren Erkrankung und die ihn beeinflussenden Faktoren dargestellt. Insbesondere wird auf differenzielle Verlaufstypen eingegangen. Das kognitive und psychosoziale Funktionsniveau sowie die psychopathologischen Besonderheiten der Erkrankung werden beleuchtet. Zudem werden biologische Faktoren und Therapieansätze herausgearbeitet, welche den Verlauf und die Prognose beeinflussen.

**Material und Methoden:**

Literaturrecherche mittels PubMed mit Fokus auf longitudinalen Studien (siehe Onlinezusatzmaterial).

**Ergebnisse:**

Bis zum aktuellen Zeitpunkt gibt es wenige Prädiktoren und Biomarker, die eine Voraussage über den Langzeitverlauf erlauben. Keiner ist ausreichend untersucht, um den klinischen Einsatz zu ermöglichen. Eine geeignete pharmakologische und psychotherapeutische Behandlung Betroffener ist unerlässlich, um erneute Krankheitsepisoden zu vermeiden.

**Diskussion:**

Der Langzeitverlauf der bipolaren Störung ist stark heterogen und facettenreich. Trotz intensiver Forschungsbemühungen sind noch keine Prädiktoren identifiziert, welche verlässlich den klinischen Verlauf vorhersagen. Umso wichtiger ist die weitere Erforschung, um individualisierte Therapieangebote zu unterbreiten, neuartige Therapien zu entwickeln und frühzeitig den Verlauf positiv zu beeinflussen.

**Zusatzmaterial online:**

Die Online-Version dieses Beitrags (10.1007/s00115-024-01791-6) enthält eine Übersicht über die verwendeten Suchbegriffe.

## Abstract (en)

**Background:**

Bipolar disorder (short: BD) is a severe illness with very heterogeneous trajectories. While some of the patients show no or hardly any long-term impairments, other affected individuals show substantial neurocognitive deficits with a clear decline in psychosocial functioning. Which factors influence the course of the disease is the subject of current research efforts.

**Objective:**

This review presents the long-term course of bipolar disease and the factors influencing it. In particular, differential trajectory types are discussed. The cognitive and psychosocial functional level as well as the psychopathological characteristics of the disease are elucidated. In addition, biological factors and treatment approaches influencing the course and prognosis are identified.

**Material and methods:**

Literature search using PubMed focusing on longitudinal studies over several years (see online supplement).

**Results:**

To date, there are only a few predictors and biomarkers that allow prediction of long-term progression. None have been sufficiently studied to enable clinical use. Appropriate pharmacological and psychotherapeutic treatment of those affected is essential to avoid renewed episodes of the disease.

**Discussion:**

The long-term course of bipolar disorder is highly heterogeneous and multifaceted. Despite intensive research efforts, no predictors have yet been identified that reliably predict the clinical course. This makes further research all the more important in order to offer individualized therapy options, develop new therapies and positively influence the course of the disease at an early stage.

Der Langzeitverlauf bipolarer Störungen (englisch: bipolar disorder, kurz: BD) ist sehr heterogen. Individuell reicht das Spektrum der Verläufe von langfristigen Remissionen mit unbeeinträchtigter Alltagsfunktionalität bis zu chronischen, unvorhersagbaren und die Lebensqualität stark einschränkenden Episoden. Faktoren, die den Verlauf beeinflussen, frühzeitig zu erkennen und eine individualisierte und adäquate Therapie zeitig einzuleiten, bleibt eine Herausforderung. In dieser Übersichtsarbeit wird der Langzeitverlauf der bipolaren Erkrankung dargestellt. Dabei wird auf differenzielle Verlaufstypen eingegangen und herausgearbeitet, welche Faktoren diese beeinflussen.

Der Begriff bipolare Störung (englisch: „bipolar disorder“, kurz: BD) umfasst eine Gruppe schwerwiegender psychischer Erkrankungen, welche mit einem chronisch-rezidivierenden Verlauf einhergehen. Dabei kommt es zu manischen bzw. hypomanischen, depressiven oder gemischten Episoden, die von beschwerdefreien euthymen Intervallen unterschiedlicher Länge unterbrochen werden. Die fünfte Auflage des Diagnostic and Statistical Manual of Mental Disorders (DSM-5) und die 11. Auflage der International Classification of Diseases (ICD-11) unterscheiden die Bipolar-I-Störung, die Bipolar-II-Störung und die Zyklothymie (Tab. 1). Bipolare Syndrome, die keinem dieser Subtypen zugeordnet werden können, werden zur Kategorie „sonstige näher bezeichnete bipolare oder verwandte Störungen“ gezählt. Alle Diagnosen eint, dass die betroffenen Individuen einen heterogenen Krankheitsverlauf aufweisen, der nicht selten mit beträchtlichen psychosozialen Funktionseinschränkungen, einem hohen Risiko für psychische Komorbiditäten und dem Verlust von 10 bis 20 Lebensjahren einhergeht ([33]; ).

**Tab. 1:** Wichtige Definitionen

Bipolar-I-Störung	Charakterisiert durch das Vorliegen von depressiven (fakultativ) und mindestens einer manischen Episode (obligat).
Bipolar-II-Störung	Charakterisiert durch das Vorliegen von depressiven und mindestens einer hypomanischen Episode. Die Patienten erreichen nie das Vollbild einer Manie.
Zyklothymie	Charakterisiert durch mehrere Episoden veränderter Stimmung während der vergangenen zwei Jahre. Diese Episoden treten nahezu kontinuierlich auf, erfüllen nach Dauer und Intensität jedoch nicht die Kriterien einer depressiven oder (hypo-)manischen Episoden.
Rapid Cycling	Definiert durch das Auftreten von mindestens vier Krankheitsepisoden unterschiedlicher Polarität pro Jahr.

## Psychosoziales Funktionsniveau

Das psychosoziale Funktionsniveau im Langzeitverlauf variiert interindividuell stark. Männliches Geschlecht, höheres Lebensalter und psychiatrische Komorbiditäten sind mit einem schlechteren funktionellen Outcome assoziiert. Ein höherer sozioökonomischer Status sowie Verheiratetsein hingegen wirken protektiv. Subsyndromal bestehende depressive Symptome werden konsistent als einer der stärksten mit einer funktionellen Beeinträchtigung assoziierten Faktoren beschrieben [35].

Dass eine BD dabei häufig mit einer Langzeitarbeitsunfähigkeit einhergeht, zeigt eine prospektive Studie, welche über 6 Jahre 152 Patienten mit BD begleitet hat. 67 (44,1 %) der Patienten erhielten am Ende eine Behindertenrente. In 54 der 67 Fälle (80,6 %) war die BD als Primärdiagnose ursächlich für die Bewilligung. Prädiktionsfaktoren für die Bewilligung waren ein älteres Alter, der BD-I-Subtyp und Komorbiditäten mit posttraumatischer Belastungsstörung, generalisierter Angststörung und vermeidender Persönlichkeitsstörung sowie die Gesamtzeit mit depressiven oder gemischten Phasen und die subjektive Arbeitsunfähigkeit zur Baseline [2].

Weitere untersuchte Variablen, die das psychosoziale Outcome beeinflussen, sind psychotische Symptome, die Episodenfrequenz, die Schlafqualität und die Erkrankungsdauer sowie neurokognitive Variablen [35, 50]. Fälschlicherweise wird der BD-Subtyp-II häufig als die weniger gravierende oder weniger chronische Verlaufsform betrachtet, dabei verbleibt es nach aktueller Studienlage umstritten, ob der BD-Subtyp einen prädiktiven Wert für einen chronischeren Verlauf hat.

## Psychopathologische Subtypen

Ein phasenweiser Wechsel depressiver, hypomaner/manischer und euthymer Episoden kennzeichnet die BD. Dabei zeichnet sich ein heterogenes Bild der psychopathologischen Beeinträchtigungen im Langzeitverlauf. Die aktuelle Datenlage deutet darauf hin, dass der kategoriale Ansatz der Einteilung, der sich in den aktuellen Klassifikationssystemen widerspiegelt, wenig Vorhersagekraft hinsichtlich des Outcomes bietet. Stattdessen weisen dimensionale Verlaufscharakteristika eine stärkere Vorhersagekraft auf.

Diesem Ansatz folgend untersuchten Uher et al. 191 Patienten mit BD zunächst über 18 Monate [57] und im Anschluss insgesamt über einen Zeitraum von 5 Jahren [58]. Sie identifizierten zwei Verlaufstypen der BD: einen episodischen Verlaufstypen und einen depressiven Verlaufstypen, welche 80 % der Verläufe Betroffener ausmachten. Die restlichen 20 % setzten sich aus unüblicheren Verlaufstypen zusammen, wie beispielsweisen Verläufen mit langwierigen Hypomanien und gemischten Episoden. Die Forscher fanden heraus, dass die Charakteristika des 18-monatigen Follow-ups das Outcome über die folgenden 3,5 Jahre voraussagten. Der Anteil des depressiven Zeitraumes, die Schwere depressiver Symptome und der Anteil des manischen Zeitraumes sagte mehr Krankheitszeit voraus. Eine höhere Wahrscheinlichkeit für Krankenhausaufenthalte wurde durch den Anteil des manischen Zeitraumes, die Schwere der manischen Symptome und einen Depression-zu-Manie-Wechsel vorhergesagt.

Auch die prädominante Polarität hat zunehmend an Wichtigkeit als Marker für den Langzeitverlauf der BD gewonnen. Es werden dabei drei Subtypen unterschieden:

- prädominant manische Polarität (MPP),
- prädominant depressive Polarität (DPP) und
- prädominant unbestimmte Polarität (IPP).

Die Zuordnung zu einem Subtyp erfolgt entweder, wenn zwei Drittel der Episoden der entsprechenden Polarität entsprechen (The Barcelona proposal [7]) oder wenn ein einfaches Überwiegen von Krankheitsepisoden einer Polarität vorliegt (The Harvard proposal [3]). Die Studienlage deutet darauf hin, dass DPP mit einer höheren Anzahl von Suizidversuchen, mehr Krankheitsepisoden, komorbiden Angststörungen und einem depressiven Beginn der Erkrankung assoziiert ist. MPP scheint hingegen mit einer höheren Rate an Substanzmissbrauch, psychotischen Symptomen, häufigeren Krankenhausaufenthalten und einem früheren sowie manischen/psychotischen Beginn einherzugehen.

Dies deckt sich überwiegend mit den Befunden einer longitudinalen Kohortenstudie, welche 87 Patienten über 7 Jahre begleitete. Die Patienten mit MPP zeigten eine höhere Rate an Krankenhausaufenthalten sowie psychotischen Symptomen. Im Gegensatz zur sonstigen Studienlage wiesen diese allerdings auch eine höhere Rate an Suizidversuchen auf und zeigten – wenn auch nicht statistisch signifikant – eine höhere Anzahl an Krankheitsepisoden. Die prädominante Polarität zur Baseline war signifikant assoziiert mit der prädominanten Polarität nach 7 Jahren [4]. Dies legt nahe, dass die Polarität im Krankheitsverlauf bipolarer Patienten gleichbleibt und damit einen starken Prädiktor des Krankheitsverlaufes darstellt.

## Kognitive Leistung

Die Untersuchung der kognitiven Funktionen bei Patienten mit BD hat an Bedeutung gewonnen, da kognitive Defizite erheblichen Einfluss auf das Alltagsleben und die Lebensqualität haben. Während es viele querschnittliche Studien mit uneinheitlichen Befunden gibt, sind Langzeitbeobachtungen rar. Einige zentrale Studien haben jedoch die langfristigen Verläufe und Subgruppen kognitiver Beeinträchtigungen untersucht.

### Stabilität und Verschlechterung kognitiver Funktionen

Flaaten et al. führten eine 10-jährige Längsschnittstudie mit 150 Patienten durch, die zur Baseline, nach 5 und 10 Jahren neuropsychologisch evaluiert wurden. Die Mehrheit der Patienten zeigte stabile kognitive Leistungen, während eine Subgruppe signifikante Verschlechterungen in Gedächtnis und Exekutivfunktionen aufwies, korreliert mit einer höheren Frequenz von Krankheitsepisoden und längerer Krankheitsdauer [11].

Ehrlich et al. identifizierten durch Clusteranalysen bei 200 Patienten über 7 Jahre drei kognitive Subgruppen: stabil, moderat beeinträchtigt und schwer beeinträchtigt. Die stabilen zeigten konstante Leistungen, die moderat Beeinträchtigten eine leichte Verschlechterung in Verarbeitungsgeschwindigkeit und Exekutivfunktionen, während die schwer beeinträchtigte Gruppe deutliche Verschlechterungen in fast allen kognitiven Domänen aufwies [9].

### Einfluss der Krankheitsepisoden und des Subtyps

Sánchez-Morla et al. untersuchten den Einfluss der Anzahl der Krankheitsepisoden auf die kognitive Entwicklung über 5 Jahre. Sie fanden heraus, dass eine höhere Anzahl von Episoden mit stärkeren Verschlechterungen in Gedächtnis und Exekutivfunktionen assoziiert ist [51]. Diese Ergebnisse bestätigen die Befunde von Flaaten et al. und Ehrlich et al.

Sleurs et al. erweiterten das Verständnis durch die Untersuchung des Einflusses des BD-Typs bei frühem Krankheitsbeginn. Sie zeigten, dass Typ I signifikante Auswirkungen auf die kognitive Funktion hat, insbesondere bei frühem Krankheitsbeginn, mit schwereren Beeinträchtigungen als bei Typ II [53].

### Zwischenfazit und Implikationen

Die aktuellen longitudinalen Studien legen nahe, dass kognitive Störungen bei BD heterogen verlaufen und verschiedene Subgruppen existieren. Während viele Patienten stabile kognitive Funktionen aufweisen, zeigen spezifische Subgruppen unterschiedliche Verläufe und Verschlechterungen, oft beeinflusst durch die Anzahl der Krankheitsepisoden und den Typ der BD.

Alle Studien betonen die Notwendigkeit einer frühzeitigen Identifikation von Patienten mit hohem Risiko für kognitive Verschlechterungen und die Entwicklung gezielter therapeutischer Interventionen, wie der kognitiven Remediation [54], die auf die spezifischen Bedürfnisse der verschiedenen Subgruppen eingehen.

## Strukturelle Hirnbildgebung

Strukturelle Veränderungen des Gehirns konnten bereits bei Personen mit genetischem Risiko für eine BD (d. h. bei Verwandten 1. Grades) gezeigt werden. Die kortikale Dicke nimmt bis zum 20. Lebensjahr rasch ab, begleitet von einer Abnahme des kortikalen Volumens. Beim Übergang ins Erwachsenenalter verlangsamt sich die Abnahmerate deutlich [12, 55]. Bei Verwandten 1. Grades wurden ein erhöhtes kortikales Volumen und eine erhöhte kortikale Dicke festgestellt [62]. Das Volumen des rechten inferioren frontalen Gyrus war am größten bei nichtbetroffenen Teilnehmern mit genetischem Risiko und korrelierte umgekehrt mit der Dauer der Erkrankung in einer kombinierten Gruppe symptomatischer Probanden mit genetischem Risiko und jungen Betroffenen mit BD [19]. Darüber hinaus zeigte eine Untersuchung des geschätzten Hirnalters mittels maschinellem Lernen keinen Unterschied zwischen Nachkommen bipolarer Eltern und gesunden Probanden [20], während bei Personen mit manifester BD (die nicht mit Lithium behandelt wurden) ein um 4,28 Jahre höheres Hirnalter festgestellt wurde [15]. Des Weiteren wurden bei Betroffenen mit manifester BD eine Verminderung der grauen Substanz beobachtet [13]. Zwei groß angelegte multizentrische Studien zeigten jeweils einen dünneren frontalen, temporalen und parietalen Kortex (*N* = 6503) [21], verminderte Volumina von Hippokampus und Thalamus sowie bei Bipolar-I-Störung zusätzlich der Amygdala (*N* = 4304; [22]). Maschinelles Lernen konnte anhand der unterschiedlichen strukturellen Muster die Betroffenen mit einer Korrektklassifikationsrate bis zu 65,23 % identifizieren [44]. Ein ähnliches Muster reduzierter kortikaler Dicke wurde bei Betroffenen mit Schizophrenie gefunden, jedoch nicht bei anderen Störungen wie Major Depression, Aufmerksamkeitsdefizit‑/Hyperaktivitätsstörung (ADHS), Zwangsstörungen oder Autismus [6]. Der Befund der verminderten grauen Substanz könnte allerdings auch bei manifester BD altersabhängig sein – eine Studie mit im Durchschnitt 10 Jahre jüngeren Betroffenen zeigte ein erhöhtes Volumen vom Putamen und keinen verminderten Kortex [56].

In Übereinstimmung mit Studien mit manifester BD, aber im Gegensatz zu den o. g. Studien mit nichtbetroffenen Personen mit genetischem Risiko, berichtet eine Studie von Mikolas et al. von jungen Erwachsenen mit einem klinischen Hochrisikoprofil über eine dünnere Hirnrinde [40]. Ein Machine-Learning-Ansatz konnte die Probanden mit höherem geschätztem Risiko mit Korrektklassifikationsrate bis zu 66,9 % stratifizieren [23, 41]. Zusammenfassend scheint es über die Lebensspanne bei Personen mit einer BD also sowohl zu Phasen mit beschleunigtem, aber auch, vorübergehend, reduziertem Abbau der kortikalen Dicke in strategischen Hirnregionen zu kommen.

## Weitere biologische Variablen

Längsschnittstudien zu biologischen Parametern bei Personen mit BD sind uneinheitlich, haben jedoch Veränderungen verschiedener biologischer, blutbasierter Parameter im Laufe der Zeit aufgezeigt, die zum gegenwärtigen Zeitpunkt aber lediglich als biologisches Signal gewertet werden können. Die Befunde unterstreichen die potenziell zukünftige Rolle als Biomarker für den Krankheitsverlauf und therapeutische neurobiologische Zielstrukturen. Die Studienlandschaft ist heterogen, und bisher haben keine biologischen Parameter ausreichende Präzision erreicht, um die Diagnose oder den Verlauf klinisch nutzbar abzubilden. Diese Studien fokussieren sich sowohl auf entzündliche als auch nichtentzündliche Marker und bieten Einblicke in die komplexe Pathophysiologie der BD.

### Entzündungsmarker

Forschungen zu Entzündungsmarkern haben konsistent deren Assoziation mit der BD gezeigt. Bei Patienten mit affektiven Störungen, einschließlich BD, konnten wiederholt erhöhte Konzentrationen von C‑reaktivem Protein (CRP) und Interleukin‑6 (IL-6) im Vergleich zu gesunden Kontrollpersonen nachgewiesen werden. Diese Marker scheinen mit der Schwere der affektiven Symptome zu korrelieren [24, 49].

Weitere Unterstützung für diese Befunde bietet eine längsschnittliche Studie, die den Zusammenhang zwischen proinflammatorischen Zytokinen und affektiven Symptomen bei BD untersuchte. Über mehrere Jahre hinweg wurden erhöhte Konzentrationen von IL‑6 und Tumornekrosefaktor α (TNF-α) signifikant mit einer Zunahme der Schwere der affektiven Episoden assoziiert, was auf deren Potenzial als Biomarker für Stimmungsschwankungen hinweist [8]. Trotz dieser vielversprechenden Ergebnisse ist die Datenlage heterogen, und es fehlt an konsistenten Nachweisen, die eine klinische Anwendung dieser Marker rechtfertigen.

### Nichtentzündliche Marker

Nichtentzündliche Biomarker wurden ebenfalls hinsichtlich ihrer longitudinalen Relevanz bei BD untersucht. Beispielsweise zeigte die Forschung zu Biomarkern des oxidativen Stresses signifikante Schwankungen in den Konzentrationen reaktiver Sauerstoffspezies (ROS) und Antioxidanzien über die Zeit. Diese Veränderungen standen in enger Verbindung mit der Schwere und Häufigkeit der affektiven Episoden, was darauf hinweist, dass oxidativer Stress eine kritische Rolle in der Pathophysiologie der BD spielen könnte [16].

Zusätzlich wurde in einer Studie über Plasmazonulin, einem Marker für die Darmpermeabilität, festgestellt, dass dessen Konzentrationen bei BD-Patienten über längere Zeiträume hinweg verändert waren. Diese Ergebnisse unterstreichen die potenzielle Rolle der Darm-Hirn-Achse bei BD, wobei Zonulinkonzentrationen in Reaktion auf affektive Episoden und Behandlungsphasen schwankten [10].

Ein weiterer interessanter, wenn auch wenig spezifischer nichtentzündlicher Marker sind die Serumspiegel des S100B-Proteins und des Myelinbasisproteins (MBP). Längsschnittstudien haben gezeigt, dass diese Marker, die mit neuronaler Schädigung und glialer Aktivierung assoziiert sind, bei BD-Patienten signifikant variieren. Erhöhte S100B- und MBP-Spiegel korrelierten mit dem Fortschreiten der affektiven Episoden und deuten auf deren Nutzen bei der Überwachung des Krankheitsverlaufs und der Therapieansprechrate hin [29]. Allerdings zeigen auch diese Ergebnisse, dass die Heterogenität der Studienergebnisse eine definitive klinische Anwendung derzeit nicht unterstützt.

Eine 3‑jährige prospektive Studie zeigt, dass BDNF(„brain derived neurotrophic factor“)-mRNA und Plasmaspiegel bei Erstdiagnose einer Major-Depression bei Patienten, die später eine bipolare Störung entwickelten, verringert sind. BDNF, entscheidend für neuronales Überleben, Wachstum und Plastizität, könnte daher perspektivisch zur früheren Erkennung von BD bei jungen Personen genutzt werden, die die Diagnosekriterien noch nicht vollständig erfüllen [32].

Ein zukunftsweisender Ansatz besteht zudem in der Identifizierung von Markern durch die Analyse des Transkriptoms aus peripherem Blut. Hier konnten bereits 26 Marker festgestellt werden, welche prognostisch relevant für den Krankheitsverlauf sind und sich im Rahmen affektiver Episoden signifikant verändern. Diese Marker gehören funktionell zu den Bereichen zirkadiane Rhythmik, Zelldifferenzierung, glutamaterge und serotonerge Signaltransduktion sowie neurotrophe Faktoren [28].

### Zwischenfazit

Longitudinalen Studien heben die facettenreiche Natur der BD hervor. Das Zusammenspiel zwischen entzündlichen Signalwegen, oxidativem Stress und neurotrophen Faktoren, die zur Neuroprogression bei BD beitragen, scheinen relevant zu sein. Entzündliche Marker wie CRP und Zytokine sind gut untersucht, nichtentzündliche Marker wie Indikatoren des oxidativen Stresses und Marker der Darmpermeabilität weniger, gewähren aber zusätzliche Einblicke in die Korrelate der Trajektorien der Erkrankung [5].

Entzündliche Marker wie CRP, IL‑6 und TNF‑α sowie nichtentzündliche Marker wie ROS, Zonulin, S100B, MBP und BDNF weisen signifikante Schwankungen auf, die mit affektiven Episoden und der Schwere der Störung im Verlauf korrelieren. Trotz vielversprechender Ergebnisse ist die Studienlandschaft heterogen und bislang kein Marker ausreichend präzise für den klinischen Einsatz. Diese Erkenntnisse unterstreichen jedoch das Potenzial dieser Biomarker zur Steuerung von Behandlungsstrategien und zur Verbesserung der prognostischen Genauigkeit bei BD.

## Einfluss von Behandlung auf den Langzeitverlauf

### Pharmakotherapie

Die Pharmakotherapie stellt die wichtigste Säule in der Langzeitbehandlung der BD dar. Ihr Ziel ist, die Anzahl und Schwere zukünftiger Episoden zu minimieren, um das Funktionsniveau und die Lebensqualität der Betroffenen möglichst langfristig zu erhalten. Frühe Stadien der Erkrankung sprechen besser auf Therapie an, weswegen eine frühzeitige Diagnose mit passender Behandlung die Prognose verbessert. Der Großteil der Patienten ist auf eine lebenslange pharmakologische Behandlung angewiesen, die standardmäßig aus einem stimmungsstabilisierenden Medikament besteht. Dieses wird entweder als Monotherapie oder in Kombination mit einem atypischen Antipsychotikum eingesetzt.

In den meisten Leitlinien wird Lithium allein oder in Kombination weiterhin als Erstlinientherapie empfohlen [18, 26, 48]. 20–30 % der bipolaren Patienten weisen eine exzellente Lithiumansprache auf [46]. Die Wirksamkeit übertrifft dabei die anderer Stimmungsstabilisatoren und die Gabe kann bei gutem Ansprechen unbegrenzt erfolgen. Dennoch ist über die letzten Jahre eine Abnahme der Verschreibungen festzustellen. Ein Grund hierfür ist die Sorge vor längerfristigen unerwünschten Arzneimittelwirkungen. Eine Kohortenstudie mit 6659 Patienten (1646 Lithiumnutzer und 5013 Referenzpatienten) über einen Zeitraum von 9,5 Jahren zeigte jedoch, dass schwere renale oder endokrine Folgen während der Lithiumbehandlung selten sind. Die mit Lithium behandelten Probanden wiesen im Verlauf abnehmende TSH(thyreoideastimulierendes Hormon)- und eGFR(„estimated“ glomeruläre Filtrationsrate)-Werte, stabile PTH(Parathormon)-Werte und steigende Kalziumspiegel auf. Zudem war die Lithiumnutzung mit höheren Raten an Nieren‑, Schilddrüsen- und Nebenschilddrüsenerkrankungen sowie ausgelenkten biochemischen Markern assoziiert, aber die absolute Anzahl an schwerwiegenden Outcomes war niedrig (z. B. chronische Nierenerkrankung: *N* = 10, 0,6 %). Zu bedenken ist, dass die Rate an Blutabnahmen bei den Lithiumpatienten deutlich höher war als bei der Referenzgruppe und Beobachtungsstudien zur Langzeitbenutzung von Lithium anfällig für Erkennungsbias sind [59].

Im Vergleich zu anderen Medikamentenklassen ist Lithium bei BD-Patienten assoziiert mit signifikant niedrigeren Suizidraten, einer geringeren Gesamtmortalität, einer verminderten Inzidenz von Krebs und Osteoporose sowie einer geringeren Demenzrate. Im Langzeitverlauf und auch bei Wiederaufnahme nach Absetzen scheint Lithium entgegen früheren Befürchtungen nicht an Effektivität einzubüßen [47].

Neben Lithium werden Antikonvulsiva wie Lamotrigin und Valproat als phasenprophylaktische Erhaltungstherapie empfohlen. Carbamazepin gehört zu den Zweitlinientherapien. Eine Metaanalyse doppelblinder, randomisierter, placebokontrollierter Studien über die Wirksamkeit und Sicherheit von Lithium und Lamotrigin in der Erhaltungstherapie klinisch stabiler Patienten mit BD zeigte, dass beide Medikamente die Rückfallrate affektiver Episoden reduzieren [45]. Valproat reduziert ebenfalls das Auftreten neuer Krankheitsepisoden und scheint dabei Lithium, atypischen Antipsychotika und anderen Antikonvulsiva nicht signifikant unterlegen [61].

Neben diesen Substanzen werden atypische Antipsychotika zur Erhaltungstherapie empfohlen. Diese Medikamente verringern die Rate an manischen Episoden, jedoch ist ihre antidepressive Wirkung uneinheitlich. Für die Kombination aus Olanzapin/Fluoxetin sowie für Quetiapin, Lurasidon und Cariprazin (entweder als Mono- oder Begleittherapie) ist die antidepressive Wirksamkeit am besten nachgewiesen [43].

Eine longitudinale Studie über 5 Jahre mit 49.298 Probanden mit BD zeigte, dass Personen, die Antipsychotika einnahmen, im Vergleich zu einer Referenzgruppe ohne Exposition gegenüber Antipsychotika eine dosisabhängige erhöhte Mortalität aufwiesen. Diese war sowohl bei den Gesamttodesursachen als auch bei den Herz-Kreislauf-Erkrankungen feststellbar. Stimmungsstabilisatoren hingegen waren unabhängig von der Dosis mit einer geringeren Gesamtmortalität verbunden. Die Autoren schlussfolgerten, dass im Zuge einer Verschreibung psychotroper Medikamente die Vorteile und mögliche unerwünschte Arzneimittelwirkungen sorgfältig miteinander abgewogen werden sollten [33].

### Pharmakotherapie ergänzen?

Bipolare Patienten müssen insbesondere bei der Langzeitanwendung von Antipsychotika abwägen, ob sie entweder die mit langfristiger Einnahme assoziierten Risiken oder ein erhöhtes Rückfallrisiko im Falle des Absetzens eingehen. Darüber hinaus kann die Pharmakotherapie allein langfristig nicht das Wiederauftreten von Stimmungsepisoden, postepisodische funktionelle Beeinträchtigungen sowie Symptome verhindern. Aus systematischen Reviews und Metaanalysen, die längsschnittliche randomisierte kontrollierte Studien (RCTs) integrieren, geht hervor, dass der kombinierte Einsatz von Pharmako- und Psychotherapie einer rein pharmakologischen Behandlung überlegen ist.

Psychotherapeutisch oftmals angewandte und effektive Verfahren sind bei BD insbesondere die kognitive Verhaltenstherapie (KVT), Psychoedukation (PE), interpersonelle und soziale Rhythmustherapie (IPSRT) und familienorientierte Therapie (FFT). Positive Einflüsse können hinsichtlich einer Symptomreduktion, Stabilisierung und Verringerung des Rückfallrisikos [38], reduzierten Rehospitalisierungsrate, Förderung der Medikamentenadhärenz [30], verlängerten Dauer bis zum Rezidiv [52], reduzierten Symptomschwere [34] sowie verbesserten psychosozialen Funktionsfähigkeit [31] erzielt werden.

### Psychotherapie

Untersuchungen, die sich mit Verläufen der BD über mehrere Jahre hinweg befassen bzw. differenzielle und verlaufsabhängige Effekte psychotherapeutischer Interventionen vergleichen, sind nach wie vor rar.

Lam et al. verglichen bei 103 stationären Patienten vom Typ I den Einfluss von KVT in Kombination mit Stimmungsstabilisatoren gegenüber deren alleiniger Gabe über 30 Monate. Diejenigen, die KVT erhielten, verbrachten im Schnitt 110 Tage weniger mit bipolaren Episoden und waren seltener von Rückfällen betroffen (64 %) als die Kontrollgruppe (84 %). Rehospitalisierungsraten sowie Zeit der Krankenhausaufenthalte waren nicht signifikant verschieden [27].

Eine Studie mit Fokus auf therapierefraktäre Patienten vom Typ I (*n* = 40) zeigte, dass Patienten mit einer Behandlungstrias aus KVT, PE und Pharmakotherapie deutlich im Vergleich zur pharmakologischen Standardbehandlung profitierten. Die Reduktion affektiver (manisch, depressiv, ängstlich) Symptome und die Verbesserung im Funktionsniveau waren über den Untersuchungszeitraum von 5 Jahren anhaltend [17].

Gruppenpsychoedukation scheint im Vergleich mit individueller PE mit einer längeren Dauer bis zur ersten Hospitalisierung assoziiert [25].

Eine Kombination aus Pharmakotherapie und PE scheint bezüglich der Zeit bis zum Rückfall, zur Genesung oder der Erkrankungsdauer der Kombination aus Pharmakotherapie und FFT nicht unterlegen. Allerdings waren Teilnehmer der familienorientierten Behandlung in der zugrunde liegenden Studie nach 2 Jahren von weniger schwerwiegenden manischen Symptomen betroffen [39].

Der Vergleich einer komplexen psychoedukativen Intervention („intense clinical management“) vs. IPSRT bei 175 Patienten vom Typ I zeigte, dass Patienten mit IPRST in der Akutphase bez. ihrer beeinträchtigten beruflichen Leistungsfähigkeit schneller remittierten (Intervention-Zeit-Interaktionseffekt). Die Verbesserung hielt zwar in der 2‑jährigen Erhaltungsphase an, war jedoch an deren Ende nicht mehr signifikant verschieden von der Vergleichsbedingung [14].

Zusammengenommen geben die Studien erste Hinweise auf langfristige, den Krankheitsverlauf günstig beeinflussende Effekte der Kombinationstherapie. Verschiedene Behandlungsleitlinien (deutsch S3, britisch NICE [National Institute for Health and Clinical Exellence], kanadisch und australisch) sprechen daher auch die Empfehlung aus, in der Stabilisierungsphase und zwecks Rezidivprophylaxe psychotherapeutische Interventionen adjunktiv anzubieten [36, 60]. Allerdings mangelt es an vergleichbaren und neueren Studien, die weiterführend Aspekte wie Sub- und Verlaufstypen berücksichtigen.

## Faktoren für die Langzeitprognose

Im Vergleich zur Normalbevölkerung weisen Patienten mit BD ein etwa doppelt so hohes Sterblichkeitsrisiko auf. Dies hängt zum einen mit einer höheren Prävalenz somatischer Erkrankungen bei dieser Patientengruppe zusammen: Beispiele hierfür sind das metabolische Syndrom, Übergewicht, Diabetes Typ 2 und Migräne. Zum anderen ist das Sterblichkeitsrisiko durch die hohe Suizidrate gesteigert (20- bis 30-fach höher als in der Allgemeinbevölkerung, höher als bei allen anderen psychiatrischen Erkrankungen [37]).

Das Suizidrisiko ist bei Patienten mit Rapid Cycling gegenüber Betroffenen ohne Rapid Cycling erhöht. Auch eine längere Krankheitsdauer und eine längerer unbehandelter Krankheitszeitraum erhöhen das Risiko eines Suizids, was auf eine höhere Frequenz und Dauer depressiver Episoden zurückgeführt wird [42].

Zudem ist die BD häufig mit psychiatrischen Komorbiditäten assoziiert, deren Vorliegen die Prognose deutlich verschlechtern. Zu diesen gehören Erkrankungen wie die Panikstörung, soziale Phobie und generalisierte Angststörung, ADHS, Persönlichkeits- sowie Essstörungen. Ein Großteil der Betroffenen leidet zeitgleich oder im Verlauf ihres Lebens an einer Alkohol- und/oder Substanzkonsumstörung [37]. Eine Traumavorgeschichte erhöht die Wahrscheinlichkeit für psychotische Symptome, Rapid Cycling, eine höhere Anzahl an Krankheitsepisoden, Suizide und Suizidversuche und beeinflusst damit den Krankheitsverlauf negativ. Ebenso verschlechtern eine geringe soziale Unterstützung sowie Saisonalität, d. h. eine regelmäßige Wiederkehr der Symptome in bestimmten Zeiträumen wie bspw. bestimmten Jahreszeiten, den Verlauf. Welchen Einfluss einschneidende Lebensereignisse haben und ob diese Folge der Erkrankung oder Beeinflussungsfaktor sind, ist bisher nicht eindeutig geklärt [1].

## Fazit für die Praxis

- Die bipolare Störungen (englisch: bipolar disorder, kurz: BD) weist eine breite Vielfalt an Verlaufsformen auf, deren beeinflussende Faktoren zahlreich und Gegenstand aktueller Forschungsbestrebungen sind.
- Am häufigsten finden sich ein episodischer Verlaufstyp mit hypomanen/manischen und depressiven Phasen, welche durch Episoden der vollständigen Remission unterbrochen sind, oder ein chronischer Verlaufstyp mit meist langanhaltend subsyndromalen depressiven Beschwerden.
- Ein Teil der Patienten weist im Langzeitverlauf keine kognitiven Einbußen auf, während ein anderer Teil zunehmende Einschränkungen i. S. einer Neuroprogression erleidet, welche sich in der Folge auch auf das psychosoziale Funktionsniveau auswirken.
- Eine genaue Kenntnis der Faktoren, welche den Krankheitsverlauf beeinflussen, ist erstrebenswert zur Durchführung frühzeitiger und individualisierter Therapieinterventionen, welche Prognose und Langzeitverlauf positiv beeinflussen.

## Supplementary Information

Übersicht über die verwendeten Suchbegriffe
