# Supplementary material for: Long-term courses of bipolar disorders
Source: Nervenarzt. 2024 Dec 21;96(1):15–22. [Article in German] doi: 10.1007/s00115-024-01791-6 (PMC11772376; doi:10.1007/s00115-024-01791-6)
Supplement: Supplementary file 1 — Übersicht über die verwendeten Suchbegriffe [file 115_2024_1791_MOESM1_ESM.pdf]

Search numb	Query	Sort By	Filters	Search Detail	Results
11	bipolar, longitudinal, prognosis			("bipolar"[All l	479
10	bipolar, longitudinal, lithium			("bipolar"[All l	324
9	bipolar, longitudinal, pharmacotherapy			("bipolar"[All l	705
8	bipolar, longitudinal, signaling mechanism			("bipolar"[All l	23
7	bipolar, longitudinal, biomarkers			("bipolar"[All l	230
6	bipolar, longitudinal, brain imaging			("bipolar"[All l	446
5	bipolar, longitudinal, cognitive neuroscien			("bipolar"[All l	124
4	bipolar, longitudinal, cognition			("bipolar"[All l	576
3	bipolar, longitudinal, psychopathology			("bipolar"[All l	265
2	bipolar, longitudinal, psychosocial functio			("bipolar"[All l	116
1	bipolar, longitudinal, course			("bipolar"[All l	812
